# Influence of the Application Time of Silane for the Bonding Performance between Feldspar or Lithium Disilicate Ceramics and Luting Resin Composites

**DOI:** 10.3390/jfb14040231

**Published:** 2023-04-19

**Authors:** Florian Fuchs, Sina Maria Westerhove, Leonie Schmohl, Andreas Koenig, Rujito Sesariojiwandono Ridho Suharbiansah, Sebastian Hahnel, Angelika Rauch

**Affiliations:** 1 Department of Prosthetic Dentistry and Material Science, Leipzig University, 04109 Leipzig, Germany; 2 Institute of Chemical Technology, Leipzig University, 04109 Leipzig, Germany; 3 Department of Dental Prosthetics, UKR University Hospital Regensburg, 93053 Regensburg, Germany

**Keywords:** silanization, etching, shear bond strength, contact angle, roughness, feldspar, lithium disilicate, surface free energy, FTIR, in vitro

## Abstract

A correct silanization time is essential for successful surface functionalization and sufficient bonding to dental ceramics. The shear bond strength (SBS) of lithium disilicate (LDS) and feldspar (FSC) ceramics and luting resin composite was investigated with respect to different silanization times, taking into account the physical properties of the individual surfaces. The SBS test was performed with a universal testing machine, and the fracture surfaces were evaluated by stereomicroscopy. The surface roughness of the prepared specimens was analyzed after etching. Changes in surface properties due to surface functionalization were evaluated by surface free energy (SFE) via contact angle measurement. Fourier transform infrared spectroscopy (FTIR) was used to determine the chemical binding. The roughness and SBS of the control group (no silane, etched) were higher for FSC than for LDS. Regarding the SFE, the dispersive fraction increased and the polar fraction decreased after silanization. FTIR confirmed the presence of silane on the surfaces. The SBS of LDS showed a significant increase from 5 to 15 s, depending on the silane and luting resin composite. For FSC, cohesive failure was observed for all samples. For LDS specimens, a silane application time of 15 to 60 s is recommended. Based on clinical conditions, no difference between the silanization times was observed for FSC specimens, indicating that etching alone produces sufficient bonding.

## 1. Introduction

The development of all-ceramic dental materials has made the adhesive cementation of fixed dental prostheses an integral part of today’s dental practice [1,2,3]. Whereas ceramics were once primarily intended for esthetical veneering of metal frameworks, they are currently commonly used as monolithic all-ceramic materials without being inferior to metal-ceramics in terms of durability [4]. Lithium disilicate ceramics (LDS) in particular have a similar long-term survival rate as metal ceramics [5], and feldspar ceramics (FSC) enable ideal esthetic adaptation to the tooth structure due to their high translucency [6]. Due to the low flexural strength of most silicate ceramics, the use of conventional cements is contraindicated; they require adhesive bonding to stabilize the ceramic framework [2,7,8]. With regard to this aspect, the adhesive procedure presents major advantages over conventional cementation, such as better bond strength, stability of the restoration, and improved esthetical behavior [3,8,9].

An important step in adhesive luting of glass ceramics is silanization [10,11,12]. Silanes produce a chemical link between the silicate ceramic surface and the polymer-based luting resin composite [13]. In addition, silanes have an impact on the surface free energy (SFE) and the associated wettability of ceramics by making hydrophilic surfaces accessible for polymer-based hydrophobic substances [14,15]. The condensation reaction of the silane with the hydroxyl groups on the inorganic ceramic surface results in covalent siloxane (-Si-O-Si-) bond formation that enables further bonding of the luting resin composite to the organic residue of the silane molecules, after which the amount of silane affects the strength of adhesion [16]. It has become obvious that the amount of silane influences the strength of adhesion. Only the layer in direct contact with the ceramic surface establishes a chemical bond with the ceramic, whereas all other layers are not stable [17,18]. Therefore, it seems possible to strengthen the bond by removing the outer layers [17]. A shortened application time of silane can result in thin layering but can lead to insufficient wetting of the ceramic surface [11]. In contrast, rapid evaporation of water and alcohol, which are bound to the surface of the ceramic by hydrogen bonding, promotes the condensation reaction to form covalent siloxane bonds [19,20].

For silane Monobond Plus (MBP; Ivoclar Vivadent, Schaan, Liechtenstein), the manufacturer recommends an application time of 60 s, whereas no application time is specified for Clearfil Ceramic Primer Plus (CCP; Kuraray Noritake, Tokyo, Japan) [21,22]. However, the application time might be a relevant factor in everyday dental practice because it might contribute to the practically simple improvement of an adhesive bond or to time savings. To address this issue, an investigation that is close to the commonly applied clinical conditions and with simple clinical feasibility would be necessary.

The aim of the present study was to investigate the shear bond strength (SBS) of two dental ceramics and luting resin composites with regard to the application time of two silanes. The bonding performance was tested by determining the shear bond strength with a universal testing machine. The surface texture was evaluated with stereomicroscopy and confocal laser scanning microscopy. Characterization of the ceramic surface at different application times of the silane was performed by analyzing the SFE on the basis of contact angle measurements. Fourier transform infrared spectroscopy (FTIR) was used to determine the bonding characteristics. The null hypotheses (H_0_) for the present study were as follows:

**H_0_(i).**  *Silane application time has no influence on SBS independent of the ceramic material or silane and luting resin composite;*

**H_0_(ii).**  *There is no linear correlation between the application time of silane and SBS.*

## 2. Materials and Methods

### 2.1. Materials

**Figure 1:**
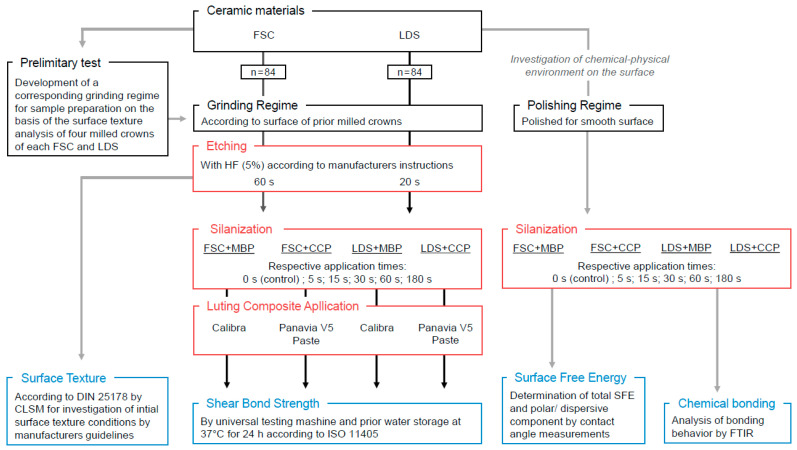
Study design with FSC (feldspar ceramic), LDS (lithium disilicate ceramic), HF (hydrofluoric acid, 5%), MBP (Monobond Plus), CCP (Clearfil Ceramic Primer Plus), SFE (surface free energy), CLSM (confocal laser scanning microscopy), FTIR (Fourier transform infrared spectroscopy).

A total of 168 specimens (Figure 1) were fabricated from FSC and LDS by cutting computer-aided design/computer-aided manufacturing (CAD/CAM) blocks into 14.0 mm × 12.5 mm × 2.3 mm slices using a precision saw (IsoMet 4000, Buehler, Lake Bluff, IL, USA). The specimens were ground under water cooling using a Pedemin-2/DAP-V semiautomatic grinding machine (Struers GmbH, Willich, Germany) following sandblasting (2.5 bar, 50 µm, 5 mm spacing, 10 s) [23]. To provide a clinically relevant study, the grinding regime of the prepared ceramic discs was adapted (P80) to the surface finish of a milled CAD/CAM restoration within a pilot study considering previous studies [24,25]. The comparative surface analysis is included in Appendix A.

The LDS specimens were crystallized (Programat P310, Ivoclar Vivadent, Schaan, Liechtenstein; T1: 830 °C for 0:10 min at 90 K/min; T2: 840 °C for 7:00 min at 30 K/min) and stored in an ultrasonic bath for 5 min. According to the manufacturer’s instructions, the ceramic surfaces were etched with hydrofluoric acid (LDS: 20 s; FSC: 60 s), rinsed with water for 60 s, and dried with oil-free air for 20 s. The two ceramics were randomly allotted to two groups and approx. a total of 0.05 mL (three droplets) of a silane was applied, either MBP or CCP. The applications were processed after 5 s, 15 s, 30 s, 60 s, or 180 s, and oil-free air was used to dry the surfaces. One group without silane (0 s) was used as a control. Seven samples per group and application duration were prepared. The luting resin composite Calibra was applied to MBP specimens, and Panavia V5 was applied to CCP specimens. For the application of the luting resin composite, prefabricated cylindrical molds with a diameter of 3 mm and a height of 3 mm were used [26]. They were polymerized for 60 s (max. 2000 mW/cm^2^, Bluephase Style 20i, Ivoclar). After removal of the mold, the specimen was polymerized again for 30 s on each side and stored in distilled water at 37 °C for 24 h [27,28]. All materials used are listed in Table 1.

### 2.2. Shear Bond Strength (SBS)

The specimens were subjected to shear bond testing in accordance with ISO/TS 11405 [28] using a universal testing machine (1446 Retroline, ZwickRoell GmbH & Co. KG, Ulm, Germany). The resin composite cylinders were positioned perpendicular to the applied force (load cell with a force limit of 500 N and a speed of 0.5 mm/min) [29,30,31]. The resulting fracture surfaces were examined by stereomicroscopy (SMZ660 8×–50×, Nikon, Tokyo, Japan), and the adhesive remnant index (ARI) [32], as well as the crack/tear-out index [33], were determined [34,35] (Table 2).

### 2.3. Surface Texture

To compare differences in the surface texture of the ceramics after etching, four different areas (100 µm × 150 µm) on 10 specimens were examined according to ISO 25178-2 [36]. A confocal laser-scanning microscope (CLSM; VK-X1000/X1050, KEYENCE, Osaka, Japan) with a 100× objective (CF IC EPI Plan SLWD; N = 0.73; WD = 4.7) and a red laser (λ = 661 nm) was used. Data analysis and filtering (L-Filter: 0.05 mm; S-Filter: 0.5 µm, filter-type: Gaussian) were performed (MultiFileAnalyzer 2.1.3.89, KEYENCE, Osaka, Japan). The arithmetical mean height (Sa/µm) was determined for the quantification of surface texture.

### 2.4. Contact Angle and Surface Free Energy (SFE)

To evaluate the SFE and surface wetting, 15 silanized specimens per silane and application time were examined. For this purpose, the ceramics were polished with diamond discs (P4000) to minimize the surface roughness and exclude topography effects in contact angle measurements. The surface roughness was validated with CLSM to ensure an Sa value of <0.02 µm. Contact angle measurements were performed using distilled water and diiodomethane (both 0.2 µL) with a DSA25S drop shape analyzer, a “Liquid Needle DO3252” dosing unit, and “ADVANCE 1.11” software (all KRÜSS GmbH, Hamburg, Germany) at 23 °C under an air atmosphere. The contact angles were measured after a delay of 30 s on both sides of the drop (fitting method: ellipse) and averaged [37,38]. Subsequently, the SFE polar components and dispersive components were calculated according to the method of Owens and Wendt [39].

### 2.5. Chemical Bonding

FTIR measurements of silanized ceramic surfaces were performed with a spectrometer (Vector 22, Bruker Corporation, Billerica, MA, USA). The spectrometer was equipped with a device for attenuated total reflectance with a diamond crystal. Spectra were recorded between 4000 and 400 cm^−1^, with a resolution of 4 cm^−1^ and 130 sample scans. Subtraction of baseline, normalization to curve area, and evaluation of peaks was performed (OPUS 3.1 Build 3, 0, 19 (20010420), Bruker Corporation, Billerica, MA, USA and OriginPro, Version 2019 OriginLab Corporation, Northampton, MA, USA).

### 2.6. Statistics

For statistical analysis (IBM SPSS Statistics 27.0.1.0, Armonk, IBM, NY, USA), the results of SBS, total SFE, polar, and dispersive components were tested for normal distribution using the Shapiro–Wilk test and for equality of variances using Levene’s test. In the case of (different) equal variances, a (Welch-) ANOVA followed by multiple comparisons for post hoc analysis with (Dunnett-T3) Bonferroni correction was performed. Pearson correlations between application time and various parameters were determined excluding the control group (no silane). To additionally test the SBS for significant differences in specimens with different silanes (MBP or CCP) at the same application time, two-tailed *t*-tests were performed, and variance inequality was accounted for by Levene’s test. The significance level was set to α = 0.05.

## 3. Results

### 3.1. Surface Texture after Etching with Hydrofluoric Acid

**Figure 2:**
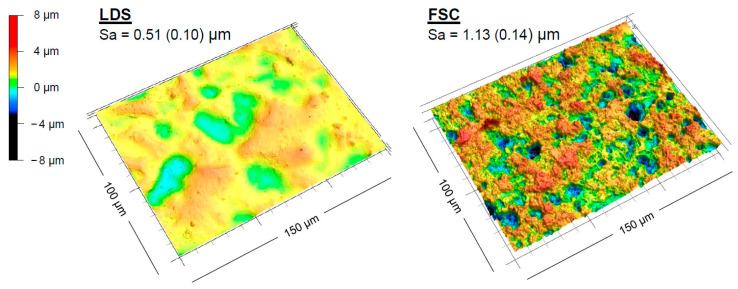
Surface texture of the initial ceramic samples after etching with hydrofluoric acid (5%) of LDS (etching time: 20 s) and FSC (etching time: 60 s) according to the manufacturer´s instructions; the arithmetical mean height (Sa) represents the surface roughness of the respective LDS or FSC group according to ISO 25178-2.

The analysis of the etched surface according to the manufacturer’s instructions revealed heterogeneous surface textures. FSC had a higher surface roughness (Sa = 1.13 µm, SD 0.14) than LDS (Sa = 0.51 µm, SD 0.10). Surface renderings presented more distinctive and deeper surface textures of FSC (Figure 2).

### 3.2. Application Times of Silane

**Figure 3:**
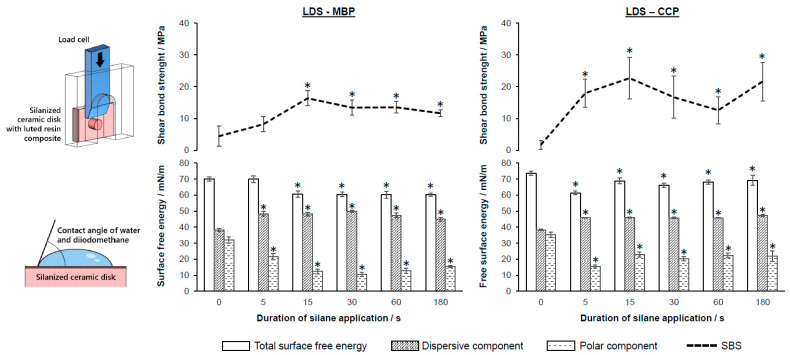
SBS of luting resin composites to LDS after application of two different silanes (MBP, Ivoclar Vi-vadent; CCP, Kuraray) as a function of the different application times (0 = control); SFE-total, polar, and dispersive components according to Owens [39]; error bars plotted as standard error; (*) significant differences (*p* < 0.05) to the control group (0 s).

LDS samples showed significant differences between the application times (Figure 3, Table 3). All specimens yielded higher SBS values (≥11.6 ± 1.1 MPa) in comparison to the control group (*p* < 0.05), except for 5 s LDS-MBP. No significant differences for LDS-MBP ≥ 15 s or LDS-CCP ≥ 5 s and its subsequent application times were observed (*p* < 0.05). Comparisons between silanes with identical application times showed higher bond strength values for LDS-CCP compared to LDS-MBP at 5 s, 15 s, and 180 s (Table 4). All LDS-MBP samples (except for the control) featured an ARI ≥ 1. Within the LDS-CCP group, all application times revealed an ARI = 1 for the majority of specimens, except for the control group. The crack/tear-out index for all LDS specimens was 0. The SFE of LDS showed a statistically significant difference from the control group at application times ≥ 5 s for LDS-MBP, and all application times for LDS-CCP. The polar fractions were decreased by silanization, whereas the dispersive fractions were increased (Figure 3). The change in the respective polar and dispersive fractions was greater than the change in SFE (see Supplementary Materials, Table S1). The SBS of LDS–MBP had a moderate correlation for SFE (*p* < 0.05; r = −0.56) and for the polar fraction (*p* < 0.05; r = −0.63) (Table 5). No other correlation was observed.

**Figure 4:**
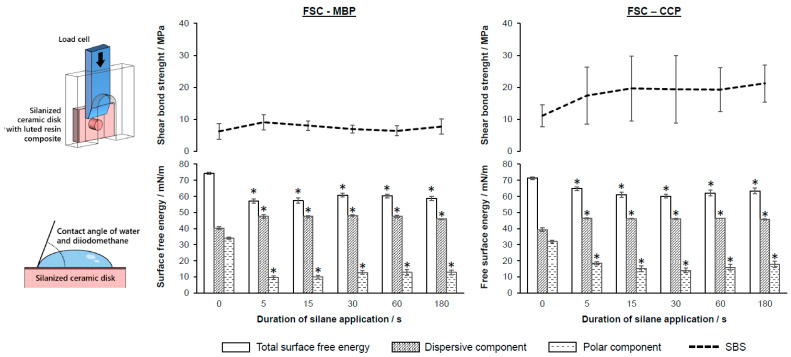
SBS of luting resin composites to FSC after application of two different silanes (MBP, Ivoclar Vi-vadent; CCP, Kuraray) as a function of the different application times (0 = control); SFE-total, polar, and dispersive components according to Owens [39]; error bars plotted as standard error; (*) significant differences (*p* < 0.05) to the control group (0 s).

The SBS of the FSC samples revealed no significant differences between the different application times independent of the silane (Figure 4, Table 3). Regarding the SBS, no significant difference between the control group and the silanized samples was determined. The ARI and crack/tear-out index presented heterogeneous results, yet all specimens showed tear-outs and/or cracks. When silane was applied, the SFE of FSC changed significantly independent of the application time. The dispersive fraction increased with an overall decrease in SFE (major changes in the polar fraction). In particular, the polar fraction was reduced at application times ≥ 5 s (Figure 4; Supplemental Material Table S1).

The investigation of the chemical bonding behavior with FTIR showed no qualitative differences regarding the application time of the silane due to overlapping peaks of the ceramics and the expected siloxane surface bonding. In contrast to the control group, the presence of silane could be detected on all samples of LDS and FSC treated with either MBP or CCP. The recorded FTIR spectra and the peak assignments are included in detail in Appendix B (Figure A2 and Figure A3, Table A1).

## 4. Discussion

In the present study, the application time of silanes for the bonding performance of LDS or FSC and luting resin composite was investigated in terms of SBS and SFE. FTIR analysis indicated the presence of silane in all samples (Figure A2 and Figure A3). This resulted in the functionalization of all material surfaces after silanization (Figure 3 and Figure 4), whereafter a change in SFE was observed, primarily attributed to a reduced polar fraction, leading to a more hydrophobic surface. Further analysis indicated a difference in the LDS and FSC surface textures that had already existed before silanization as a result of hydrofluoric etching, with the increased roughness of the FSC compared to the LDS resulting in a higher Sa value (Figure 2).

**H_0_(i):**  *Silane application time has no influence on SBS independent of the ceramic material, luting resin composite, or silane.*

The null hypothesis (i) was partially rejected. Silanization of LDS at different times showed a significant difference from the control group in the resulting SBS, after which higher values resulted for the application time after a minimum of 15 s with MBP (≥11.6 ± 1.1) and after just 5 s with CCP (≥12.6 ± 4.3). Despite the SBS measurements, the ARI revealed different results depending on the application time. The SBS yielded significantly higher values with LDS-CCP compared to LDS-MBP at silanization times of 5 and 15 s. Moreover, for the maximum application time (180 s), significantly higher bond strength values were again observed for the LDS-CCP group, although a partial ARI of 0 (no resin composite on the ceramic surface) was recorded, indicating reduced bond strength. In addition, longer application times within the LDS-MBP group resulted in higher ARI values. For FSC, no significant changes in SBS depending on application time were observed. Based on the crack/tear-out index, it must be assumed that for all FSC groups (including control), cohesive failure of the ceramic occurs.

Previous studies have shown analogous trends and similar values. Specifically, the SBS was reported by Mokhtarpour et al. (2017) to be 10.52 ± 5.39 MPa for LDS and 5.96 ± 3.66 MPa for FSC [30]. Even though the SBS for FSC differs, a predominantly cohesive failure with respect to the failure modulus was also observed in this case. Moreover, in the study of Kitahara et al. (2013), the same SBS values (14.94 ± 4.28 MPa) were also obtained for the silanization of LDS with CCP at an application time of 60 s [31]. Under a comparable preparation regime following CAD/CAM milled specimens, Straface et al. (2019) found higher SBS of LDS (8.1 ± 1.0 MPa) compared to FSC (7.2 ± 0.9) [40]. However, according to the authors, there was a higher arithmetical mean height of the surface (Ra = 1.88–2.71 µm) than in the present study (Sa = 0.51–1.13 µm), which could result in larger SBS due to higher retention forces. Compared to the previously mentioned studies, the study by Kamada et al. (2006) showed far higher values for the SBS of the investigated FSC (45.9 ± 1.6 MPa) using the same silane [41]. Although no indicator for a cause of such a deviating result can be identified, the authors also documented exclusively cohesive failure or crack propagation in the tested ceramic. The research of El-Damanhoury and Gaintantzopoulou (2017) also showed higher SBS (LDS: 37.60 ± 10.68 MPa, FSC: 27.97 ± 6.38 MPa) but also a trend of higher determined SBS for the LDS group compared to the FSC group as well as an occurrence of cohesive failure of the FSC at 80% [42]. Hu et al. (2016) initially determined an equal surface texture of the investigated samples with Ra = 1.41 ± 0.10 µm after etching for FSC. Nevertheless, higher SBS values of 27.8–32.0 MPa were found but also a 70% occurrence of cohesive failure [43]. According to El Mourad (2018), the difference in the higher SBS values occurring in these two could be attributed, for instance, to a smaller sample geometry of the luting resin composite, which increases with a smaller bonding surface [44]. Thus, from the results of this, as well as previous studies, the mechanical strength of the feldspar ceramic can be identified as the critical factor for the frequent failure in the setting of an SBS test, which is due to the lower flexural strength (biaxial) of the FSC with 120 MPa compared to the LDS with 610–650 MPa [45].

Furthermore, due to the clinically based study design, even in the absence of a silane coupling agent, a strong bonding performance for FSC could be observed, which can be explained by the increased surface roughness compared to the LDS surface after etching (Figure 2). Without silanization, the higher SBS values of FSC (6.2–11.1 MPa) compared to LDS (1.7–4.4 MPa) and the tear-outs/cracks visible in the surface of FSC indicate that increased retention of the luting resin composite due to the etching process plays a crucial role in a sufficient adhesive bond.

**Figure A1:**
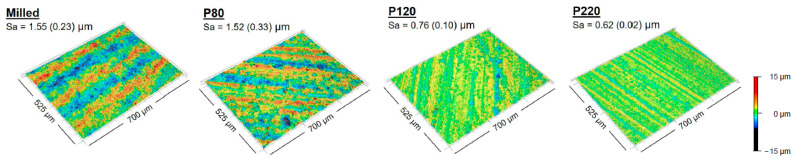
Milled and grinded (P80, P120, P220) CAD/CAM (feldspar) ceramic blocks in comparison of surface roughness by the arithmetical mean height (Sa).

**Figure A2:**
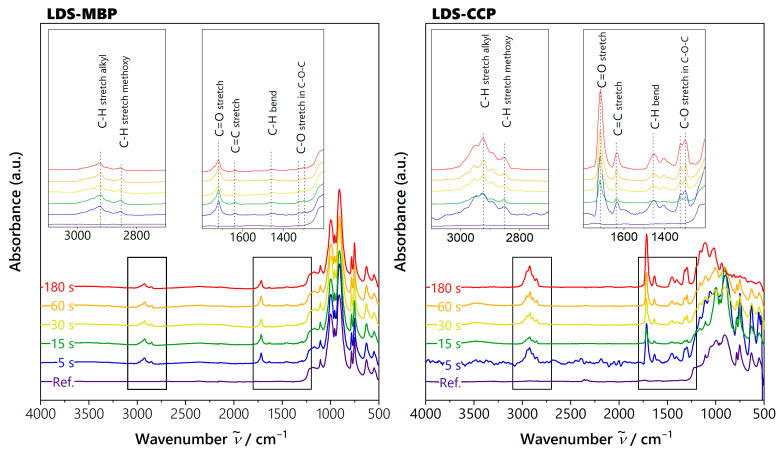
FTIR spectra of lithium disilicate ceramic (LDS) with different application times of silane; Monobond Plus (MBP) or Clearfil Ceramic Primer Plus (CCP).

**Figure A3:**
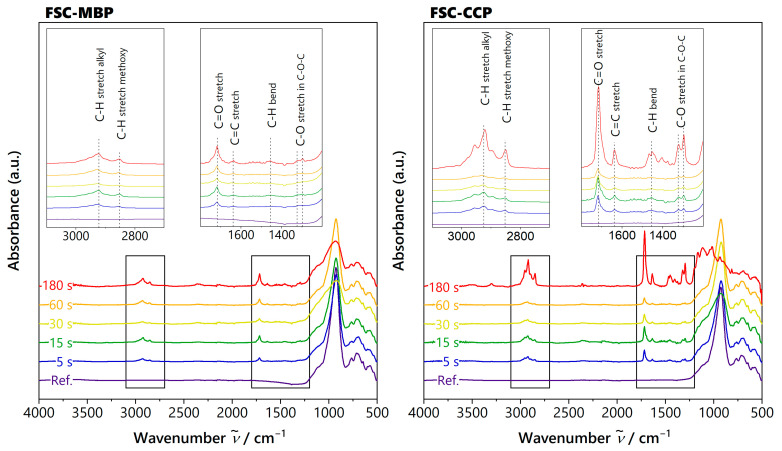
FTIR spectra of feldspar ceramic (FSC) with different application times of silane; Monobond Plus (MBP) or Clearfil Ceramic Primer Plus (CCP).

These observations are in contrast to the study of Brentel et al. (2006), who found a difference with the nonsilanized control group; however, despite a smaller bonding area, they obtained similar SBS values (8.9 ± 3.1 MPa) [10]. This discrepancy with the results of the comparison with the control group can be attributed to the different preparation protocols, which, with the adjustment of the grinding regime in the present study, closely resemble a real clinical environment where milled restorative surfaces are expected (see Figure A1).

A more advanced method to strengthen the bond between ceramic and luting resin composite by orienting and linking the silane layers is a subsequent dry heat treatment, which was already recommended by prior studies [46,47]. In addition, rinsing with warm water was not recommended. The extent to which this can be used to represent a clinical setting is yet to be determined because there is limited time capacity between try-in and insertion of a restoration during dental visits. To provide a link to clinical implementation, it is recommended that practical conditions be taken into account in future studies.

**H_0_(ii).**  *There is no linear correlation between the application time of silane and SBS.*

The null hypothesis (ii) was accepted. No significant correlations between the application time and the SBS for almost all specimens were observed. Nevertheless, this assumption is based on the fact that the Pearson correlation only provides information about a linear relationship. A nonlinear correlation cannot be excluded. However, a correlation between the total SFE, or the polar component, and the SBS could be demonstrated for the application of MBP on LDS (Table 5). Silanization has the strongest influence on the polar component of the SFE, after which it decreases and the surface becomes more hydrophobic, which also reduces the total SFE (Figure 3 and Figure 4). In the case of the LDS samples, a coexistence of this reduction of the polar component with an increased adhesion could be observed.

As a limiting factor for the present study design, no quantitative conclusions on the bonding between the silane and the ceramic could be drawn from FTIR analyses. In addition, due to the clinically oriented study design, silane residues were identified on the ceramic surface and might negatively influence SBS. Furthermore, a possible failure of the resin composite material, in the form of a predetermined breaking point due to damage produced during the removal of the mold, cannot be excluded [11]. Because of potential adhesive and/or cohesive failures, SBS investigations may provide unreliable results. Thus, it is possible that SBS analyses do not yield a fully comprehensive overview of the bond strength and its clinical behavior [43], which is why additional tensile bond strength approaches are recommended [44,48]. In the present in vitro investigation, the ceramic specimens were processed in accordance with the manufacturer’s instructions. An influence of further crystallization and related potential changes in the microstructure or the ratio between the amorphous and crystalline fractions of the materials might be possible. Furthermore, the study is limited to FSC and LDS, as well as the particular silanes and luting resin composites used. Other materials with varying compositions should be considered in terms of the SBS and surface functionalization. The connective area between the ceramic and the bonding surface, the time and intensity of light curing, and other influencing factors, such as plaque or saliva and long-term studies [29], should also be evaluated in future investigations. In addition, bonding should be evaluated after a fatigue period in a clinical setting. Therefore, in addition to static test setups, cyclic loading tests both with respect to thermal aging [46] and in combination with mechanical loading, for example, by chewing simulation [49], are of further interest for the effects on the composite after accelerated aging. It is recommended that additional approaches to improve silanization with emphasis on clinical applicability should be investigated, such as residue removal with water or air at elevated temperatures [14,19,20,47]. In this context, a comprehensive evaluation of the bonding behavior by FTIR as well as the investigation of the functionalized surface SFE analysis is recommended, as described in the present study or done by previous research groups [50].

## 5. Conclusions

The bond strength is partly dependent on the application time of the silane coupling agent. For lithium disilicate ceramics, a silanization time between 15 and 60 s produced the best possible bonding behavior in terms of SBS, with the addition that silanization with CCP produced higher bond strengths at low application times. For clinical application, silanization times should not be shorter than 15 s, whereas silanization times longer than 60 s do not necessarily result in increased adhesion forces. After conditioning with hydrofluoric acid, the silanization process seems to have only a minor influence on feldspar ceramics since the strongly distinctive surface affects the bond strength by retention. Thus, the etching of a feldspar ceramic is a crucial step for achieving a sufficient bond with the luting resin composite. The analysis of the SFE revealed significant differences due to the functionalization of the surface, after which the dispersive component was increased, whereas the polar component was decreased to a greater extent, resulting in an overall decrease in the total SFE. Apart from confirming the presence of silane on the surfaces, FTIR was not suitable for analyzing the samples in this clinically adapted study design.

## Figures and Tables

**Table 1:** Materials used in the present study.

	Material/Ident.	Product	Manufacturer	LOT
Ceramics	Feldspar FSC	Vitablocs Mark II A2C, I-14	Vita Zahnfabrik Bad Säckingen, Germany	79391 73530
	Lithium disilicate LDS	IPS e.max CAD LT A2, C14	Ivoclar Vivadent Schaan, Liechtenstein	W86607 Z00S50
Silanes	MBP	Monobond Plus	Ivoclar Vivadent Schaan, Liechtenstein	X30882 Y39578
	CCP	Clearfil Ceramic Primer Plus	Kuraray Noritake Dental Inc. Chiyoda, Japan	6K0036 9R0056
Surface Conditioning	Hydrofluoric acid 5%	IPS Ceramic Etching Gel	Ivoclar Vivadent Schaan, Liechtenstein	X39271 Y37000
Luting resin composite	For MBP Specimen	Calibra translucent	Dentsply DeTrey GmbH Konstanz, Germany	00041643 00023656
	For CCP Specimen	Panavia V5 Automix (Intro Kit) clear	Kuraray Noritake Dental Inc. Chiyoda, Japan	000098

**Table 2:** Classification of resulting fracture surfaces.

Score	Adhesive Remnant Index (ARI) [32]	Crack/Tear-Out Index [33]
0	No resin composite on the ceramic surface	No cracks or tear-outs visible in ceramic surface
1	<50% resin composite on the ceramic surface	Cracks visible in ceramic surface
2	>50% resin composite on the ceramic surface	Tear-outs visible in ceramic surface
3	Ceramic surface largely covered with resin composite	Cracks and tear-outs visible in ceramic surface

**Table 3:** Mean SBS, crack/tear-out index, and ARI results of all tested specimens for LSD and FSC for each application time of the silane coupling agents MBP and CCP; capital letters indicate significant differences between silane application times within the respective group; SD: standard deviation; 95%-CI: confidence interval.

Material and Silanization			SBS/MPa			Crack/Tear-Out/%	ARI/%
Group	Silanization Time	Code	Mean ± SD	95%-CI	*p* < 0.05	0; 1; 2; 3	0; 1; 2; 3
LDS-MBP	0	0	4.4 ± 3.2	(1.2; 7.7)	BCDE	100; 0; 0; 0	43; 57; 0; 0
	5	A	8.2 ± 2.4	(5.8; 10.6)	BCD	100; 0; 0; 0	0; 57; 14; 29
	15	B	16.3 ± 2.4	(13.9; 18.7)	0AE	100; 0; 0; 0	0; 100; 0; 0
	30	C	13.4 ± 2.4	(11.1; 15.8)	0A	100; 0; 0; 0	0; 71; 29; 0
	60	D	13.5 ± 1.9	(11.6; 15.4)	0A	100; 0; 0; 0	0; 57; 43; 0
	180	E	11.6 ± 1.1	(10.5; 12.8)	0B	100; 0; 0; 0	0; 57; 43; 0
LDS-CCP	0	0	1.7 ± 1.4	(0.3; 3.1)	ABCDE	100; 0; 0; 0	100; 0; 0; 0
	5	A	18.0 ± 4.5	(13.5; 22.4)	0	100; 0; 0; 0	0; 100; 0; 0
	15	B	22.7 ± 6.6	(18.9; 34.0)	0D	100; 0; 0; 0	0; 100; 0; 0
	30	C	16.7 ± 6.6	(11.8; 27.1)	0	100; 0; 0; 0	0; 100; 0; 0
	60	D	12.6 ± 4.3	(9.7; 20.0)	0B	100; 0; 0; 0	0; 100; 0; 0
	180	E	21.6 ± 6.0	(15.1; 28.0)	0	100; 0; 0; 0	14; 86; 0; 0
FSC-MBP	0	0	6.2 ± 2.5	(3.8; 8.7)	*-*	0; 0; 100; 0	71; 29; 0; 0
	5	A	9.1 ± 2.3	(6.7; 11.4)	*-*	14; 0; 43; 43	57; 29; 14; 0
	15	B	8.1 ± 1.5	(6.6; 9.5)	*-*	0; 0; 100; 0	0; 100; 0; 0
	30	C	6.9 ± 1.3	(6.7; 9.5)	*-*	0; 0; 100; 0	0; 100; 0; 0
	60	D	6.4 ± 1.5	(4.8; 7.9)	*-*	57; 0; 43; 0	43; 57; 0; 0
	180	E	7.7 ± 2.3	(5.2; 10.2)	*-*	14; 0; 86; 0	14; 57; 14; 0
FSC-CCP	0	0	11.1 ± 3.4	(7.7; 14.5)	*-*	14; 0; 29; 57	100; 0; 0; 0
	5	A	17.4 ± 8.9	(8.5; 26.3)	*-*	0; 0; 71; 29	100; 0; 0; 0
	15	B	19.7 ± 10.1	(9.6; 29.8)	*-*	0; 0; 57; 43	100; 0; 0; 0
	30	C	19.4 ± 10.5	(8.9; 29.9)	*-*	14; 0; 57; 29	57; 14; 14; 0
	60	D	19.3 ± 6.9	(12.4; 26.2)	*-*	29; 29; 29; 14	29; 43; 14; 14
	180	E	21.3 ± 5.8	(15.0; 27.4)	*-*	0; 0; 57; 43	43; 57; 0; 0

**Table 4:** Result of the two-tailed *t*-test to evaluate the significant difference for the SBS of the groups of the LDS silanized with MBP or CCP at the respective silane application times; the column “Out-come” provides an interpretation of the *t*- and *p*-values.

Application Time	*t*-Value	*p*-Value	Outcome
0	1.891	0.083	
5	−4.721	0.000	SBS(MBP) < SBS(CCP)
15	−3.473	0.005	SBS(MBP) < SBS(CCP)
30	−1.933	0.101	
60	−0.561	0.593	
180	−3.727	0.009	SBS(MBP) < SBS(CCP)

**Table 5:** Pearson correlation coefficients (r) and *p*-values for the SBS and the parameters: silane application time; total SFE, polar component, and dispersive component of LDS and FSC, silanized with MBP or CCP.

Group			Application Time	Total SFE	Polar Component	Dispersive Component
LDS	MBP	r	−0.06	−0.56	−0.63	0.09
		*p*-Value	0.724	<0.001	<0.001	0.592
	CCP	r	0.03	0.18	0.17	0.10
		*p*-Value	0.857	0.315	0.348	0.574
FSC	MBP	r	−0.15	−0.29	−0.24	−0.01
		*p*-Value	0.406	0.096	0.171	0.584
	CCP	r	0.11	0.04	0.04	−0.00
		*p*-Value	0.514	0.836	0.842	0.997

## Data Availability

The datasets generated during and/or analyzed during the current study are available from the corresponding authors on reasonable request, unless already presented in the article, included in the Appendix A and Appendix B or in the Supplementary Materials.

## Supplementary Materials

The following supporting information can be downloaded at: https://www.mdpi.com/article/10.3390/jfb14040231/s1, Table S1. the results for surface free energy, dispersive, and polar component of all tested groups after silanization and statistical evaluation.

## Appendix A

In order to provide a clinically relevant study, the grinding regime of the prepared ceramic discs was adapted to the surface finish of a milled CAD/CAM restoration in a pilot study and in comparison to previous studies of Mota et al. (2017) with an Sa = 1.8 µm and Flury et al. (2012) with Sa = 1.68 µm [24,25]. For this purpose, restorations made of LDS and FSC were processed by using a milling machine (InLab MC XL, Dentsply Sirona, York, PA, USA) and examined for surface texture with confocal laser scanning microscopy (CLSM) with a 20× objective (CF IC EPI Plan 20×, N = 0.46; WD = 3.1). Based on the processed surface texture analysis (F-Filter: 0.25 mm; S-Filter: 0.8 µm, filter type: Gaussian), the preparation regime of the ceramic discs was adjusted and validated for all fabricated specimens with CLSM analysis.

## Appendix B

FTIR measurements of silanized surfaces and Peak assignment of FTIR spectra:

**Table A1:** Peak assignment of FTIR spectra (v…stretching vibration mode, δ…bending vibration mode); Monobond Plus (MBP); Clearfil Ceramic Primer Plus (CCP).

Wave Number $\tilde{\nu }$/cm^−1^	Silane Coupling Agent	Ceramic	Ref.
**Silane Coupling Agent Vibrations in MBP and CCP**			
2924	ν(C-H) in -CH_2_-/-CH_3_		[51,52]
2852	ν(C-H) in -OCH_3_		[51,52]
1717	ν(C=O)		[51,52,53]
1638	ν(C=C)		[51,52]
1455	δ(C-H)		[52,53]
1324	ν(C-O) in C-O-C		[51,53]
1297	ν(C-O) in C-O-C		[51,53]
**Lithium disilicate ceramic and potential overlapping silane vibration modes**			
1256–1185 (shoulder)	P=O [MBP]	ν(Si-O-Si)	[54,55]
1171	ν(P-O), ν(Si-O-CH_3_) [MBP, CCP]	ν(Si-O-X) (X=Zr/Ti)	[47,55,56]
1107	ν(P-O) [MBP]	ν(Si-O), ν(Si-O-C)	[52,57,58]
1001	ν(P-O) [MBP]	ν(Si-O-Si)	[53,59]
959		Si-O strong bonds	[47]
910	Si-OH [MBP, CCP]	ν(Si-O)	[51,60]
785		ν(Si-O-Si) in lithium disilicate	[61]
754		ν(Si-O-Si) in lithium disilicate	[61]
631		ν(Si-O-Si) in lithium disilicate	[61]
552		δ(O-Si-O) in lithium disilicate	[61]
**Feldspar ceramic and potential overlapping silane vibration modes**			
1273–995 (overlapping shoulders)	Si-O-Si, ν(Si-O-C), P-O-C [MBP, CCP]	ν(Si-O), ν(Al-O)	[51,58,59,60]
924		ν(Si-O), ν(Al-O)	[47,58,60]
908 (shoulder)	Si-OH [MBP, CCP]	ν(Si-O), ν(Al-O)	[51,56,58,60]
766		ν(Si-O-Si), ν(Al-O-Si), δ(Si-O-Si)	[62]
718		Aluminium and silicon rings	[58]
648		Aluminium and silicon rings	[58]
602		ν(Si-O-Si)	[61]
576		Aluminium and silicon rings	[58,60]
538		δ(O-Si-O), δ(Si-O-Si)	[61,62]
