# Supplementary material for: Influence of the Application Time of Silane for the Bonding Performance between Feldspar or Lithium Disilicate Ceramics and Luting Resin Composites
Source: J Funct Biomater. 2023 Apr 19;14(4):231. doi: 10.3390/jfb14040231 (PMC10143910; doi:10.3390/jfb14040231)
Supplement: Supplementary file 1 [file jfb-14-00231-s001.zip › jfb-2295908-SI.pdf]

The application time of silane influences the adhesive bond between silicate ceramics and luting resin composites

Surface free energy of all tested groups after silanization

Table S1. Results for surface free energy, dispersive and polar component as mean of all tested specimen for lithium disilicate ceramic (LDS) and feldspar ceramic (FSC) for each application time of the silane coupling agents Monobond Plus (MBP) and Clearfil Ceramic Primer Plus (CCP); capital letters indicate significant differences between silane application times within the respective group; SD: standard deviation; 95%-CI: confidence interval.

Material and Silanization			Dispersive component / mN/m			Polar component / mN/m			Total SFE / mN/m		
**Group**	**Silan. / s**	**Code**	**Mean ± SD**	**95%-CI**	***p* < 0.05**	**Mean ± SD**	**95%-CI**	***p* < 0.05**	**Mean ± SD**	**95%-CI**	***p* < 0.05**
LDS-MBP	0	0	38.2 ± 0.9	(37.3; 39.0)	*ABCDE*	32.0 ± 1.9	(30.3; 33.7)	*ABCDE*	70.1 ± 1.3	(69.0; 71.3)	*BCDE*
	5	A	48.4 ± 1.3	(47.1; 49.6)	*0 E*	21.5 ± 2.0	(19.7; 23.3)	*0BCDE*	69.8 ± 2.2	(67.8; 71.9)	*BCDE*
	15	B	48.2 ± 1.1	(46.8; 49.1)	*0 E*	12.4 ± 1.6	(10.5; 14.1)	*0AE*	60.5 ± 2.0	(58.1; 62.4)	*0A*
	30	C	49.8 ± 0.6	(49.1; 50.4)	*0DE*	10.5 ± 1.2	(9.1; 11.9)	*0AE*	60.3 ± 1.4	(58.6; 61.9)	*0A*
	60	D	47.4 ± 1.6	(45.5; 48.9)	*0CE*	12.8 ± 1.5	(11.6; 12.9)	*0AE*	60.1 ± 2.1	(58.2; 60.7)	*0A*
	180	E	44.9 ± 1.2	(43.8; 46.0)	*0ABCD*	15.4 ± 0.7	(14.7; 16.1)	*0ABCD*	60.3 ± 1.0	(59.3; 61.2)	*0A*
LDS-CCP	0	0	38.3 ± 0.5	(37.9; 38.7)	*ABCDE*	35.2 ± 1.7	(33.6; 36.7)	*ABCDE*	73.5 ± 1.4	(72.2; 74.7)	*ABCDE*
	5	A	45.9 ± 0.1	(45.8; 46.0)	*0E*	15.5 ± 1.0	(14.5; 16.4)	*0BCDE*	61.4 ± 1.1	(60.3; 62.4)	*0BCDE*
	15	B	45.9 ± 0.2	(45.7; 46.1)	*0E*	22.9 ± 1.7	(21.1; 24.7)	*0A*	68.8 ± 1.8	(66.9; 70.8)	*0A*
	30	C	45.7 ± 0.4	(45.3; 46.2)	*0E*	20.4 ± 1.3	(19.1; 21.7)	*0A*	66.1 ± 1.3	(64.8; 67.5)	*0A*
	60	D	45.7 ± 0.2	(45.6; 45.9)	*0E*	22.3 ± 1.5	(20.8; 23.9)	*0A*	68.1 ± 1.4	(66.6; 69.5)	*0A*
	180	E	47.3 ± 0.6	(46.8; 47.8)	*0ABCD*	21.9 ± 3.2	(19.0; 24.8)	*0A*	69.2 ± 3.2	(66.3; 72.1)	*0A*
FSC-MBP	0	0	40.2 ± 0.8	(39.5; 41.0)	*ABCDE*	34.0 ± 0.6	(33.5; 34.5)	*ABCDE*	74.2 ± 0.7	(73.6; 74.8)	*ABCDE*
	5	A	47.6 ± 1.2	(46.5; 48.7)	*0E*	9.5 ± 1.0	(8.5; 10.5)	*0CDE*	57.1 ± 1.4	(55.8; 58.4)	*0CD*
	15	B	47.5 ± 0.7	(46.7; 48.0)	*0E*	9.8 ± 1.3	(8.5; 10.5)	*0CDE*	57.3 ± 1.7	(55.5; 58.2)	*0CD*
	30	C	48.1 ± 0.5	(47.6; 48.6)	*0E*	12.7 ± 1.1	(11.5; 13.9)	*0AB*	60.8 ± 1.0	(59.7; 61.8)	*0AB*
	60	D	47.6 ± 0.8	(46.6; 48.5)	*0E*	12.7 ± 1.7	(10.8; 14.7)	*0AB*	60.3 ± 1.1	(59.0; 61.6)	*0AB*
	180	E	46.0 ± 0.1	(45.8; 46.1)	*0ABCD*	12.8 ± 1.4	(11.4; 14.1)	*0AB*	58.7 ± 1.5	(57.4; 60.1)	*0*
FSC-CCP	0	0	39.3 ± 1.0	(38.4; 40.3)	*ABCDE*	31.9 ± 0.9	(31.1; 32.7)	*ABCDE*	71.2 ± 0.7	(70.6; 71.9)	*ABCDE*
	5	A	46.3 ± 0.3	(46.0; 46.5)	*0E*	18.5 ± 1.1	(17.5; 19.5)	*0BCD*	64.7 ± 1.0	(63.8; 65.7)	*0BCD*
	15	B	46.1 ± 0.2	(45.9; 46.2)	*0E*	15.0 ± 1.7	(13.3; 17.1)	*0AE*	61.1 ± 1.6	(59.4; 63.1)	*0A*
	30	C	46.2 ± 0.3	(45.8; 46.5)	*0E*	14.0 ± 1.4	(12.8; 15.6)	*0AE*	60.1 ± 1.2	(59.2; 61.5)	*0AE*
	60	D	46.2 ± 0.1	(46.2; 46.3)	*0E*	15.8 ± 1.8	(13.8; 17.9)	*0A*	62.1 ± 1.8	(60.0; 64.1)	*0A*
	180	E	45.7 ± 0.2	(45.5; 45.9)	*0ABCD*	17.7 ± 2.0	(15.9; 19.5)	*0BC*	63.4 ± 1.9	(61.7; 65.1)	*0C*
